# Restricted activity and persistent pain following motor vehicle collision among older adults: a multicenter prospective cohort study

**DOI:** 10.1186/s12877-016-0260-z

**Published:** 2016-04-19

**Authors:** Timothy F. Platts-Mills, Robert J. Nicholson, Natalie L. Richmond, Kushang V. Patel, Eleanor M. Simonsick, Robert M. Domeier, Robert A. Swor, Phyllis L. Hendry, David A. Peak, Niels K. Rathlev, Jeffrey S. Jones, David C. Lee, Mark A. Weaver, Francis J. Keefe, Samuel A. McLean

**Affiliations:** Department of Emergency Medicine, University of North Carolina, 170 Manning Dr, Chapel Hill, NC USA; Department of Anesthesiology and Pain Medicine, University of Washington, 1959 NE Pacific St, Seattle, WA USA; Intramural Research Program, National Institute on Aging, 251 Bayview Boulevard, Suite 100, Baltimore, MD USA; Department of Emergency Medicine, Saint Joseph Mercy Health System, 5301 McAuley Drive, Ypsilanti, MI USA; Department of Emergency Medicine, William Beaumont Hospital, 3601 W 13 Mile Rd, Royal Oak, MI USA; Department of Emergency Medicine, University of Florida College of Medicine Jacksonville, 1515 SW Archer Road, Gainesville, FL USA; Department of Emergency Medicine, Massachusetts General Hospital, 55 Fruit St, Boston, MA USA; Department of Emergency Medicine, Baystate Medical Center, 759 Chestnut Street, Springfield, MA USA; Department of Emergency Medicine, Spectrum Health – Butterworth Campus, 100 Michigan St. NE, 49503 Grand Rapids, MI USA; Department of Emergency Medicine, North Shore University Hospital, 300 Community Dr, Manhasset, NY USA; Department of Medicine, University of North Carolina, 170 Manning Dr, Chapel Hill, NC USA; Department of Psychiatry, Duke University, Durham, 17 Chapel Drive, Durham, NC USA; Department of Anesthesiology, University of North Carolina, 170 Manning Dr, Chapel Hill, NC USA

**Keywords:** Aged, Motor Activity, Emergency medicine, Pain, Geriatrics, Traffic accidents

## Abstract

**Background:**

Restricted physical activity commonly occurs following acute musculoskeletal pain in older adults and may influence long-term outcomes. We sought to examine the relationship between restricted physical activity after motor vehicle collision (MVC) and the development of persistent pain.

**Methods:**

We examined data from a prospective study of adults ≥65 years of age presenting to the emergency department (ED) after MVC without life-threatening injuries. Restricted physical activity 6 weeks after MVC was defined in three different ways: 1) by a ≥25 point decrease in Physical Activity Scale in the Elderly (PASE) score, 2) by the answer “yes” to the question, “during the past two weeks, have you stayed in bed for at least half a day?”, and 3) by the answer “yes” to the question, “during the past two weeks, have you cut down on your usual activities as compared to before the accident?” We examined relationships between each definition of restricted activity and pain severity, pain interference, and functional capacity at 6 months with adjustment for confounders.

**Results:**

Within the study sample (*N* = 164), adjusted average pain severity scores at 6 months did not differ between patients with and without restricted physical activity based on decreased PASE score (2.54 vs. 2.07, *p* = 0.32). In contrast, clinically and statistically important differences in adjusted average pain severity at 6 months were observed for patients who reported spending half a day in bed vs. those who did not (3.56 vs. 1.91, *p* < 0.01). In adjusted analyses, both decreased PASE score and cutting down on activity were associated with functional capacity at 6 months, but only decreased PASE score was associated with increased ADL difficulty at 6 months (0.70 vs. -0.01, *p* = 0.02).

**Conclusions:**

Among older adults experiencing MVC, those reporting bed rest or reduced activity 6 weeks after the collision reported higher pain and pain interference scores at 6 months. More research is needed to determine if interventions to promote activity can improve outcomes after MVC in older adults.

## Background

Chronic musculoskeletal pain causes more disability globally than any other condition [1] and is the single most costly medical problem in the United States [2]. Chronic pain is particularly common and problematic among older adults, with more than half of community dwelling adults aged 65 years and older reporting pain in the past month and more than one third reporting pain that interferes with daily activities [3, 4]. Chronic pain also increases older adults’ risk of functional decline, falls, and reduced life expectancy [5–8]. Thus, preventing chronic pain among older adults is an important public health priority.

One mechanism leading to chronic pain in older adults is motor vehicle collisions (MVCs). MVCs are the second most common type of trauma among older adults and result in an estimated 250,000 emergency department (ED) visits in the United States annually [9]. Due to demographic changes, the number of older adults experiencing an MVC is expected to double over the next 2 decades [10]. Persistent pain after MVC, most often in the neck and back, commonly occurs in individuals of all ages but is especially common among older adults [9, 11–14]. As with other musculoskeletal pain conditions, persistent pain after MVC places older adults at increased risk for functional decline and is also associated with reduced self-rated health and a change in living situation to obtain additional help [14, 15]. Despite the growing burden of post-MVC pain, the etiology of persistent pain after MVC among older adults remains poorly understood, and there is no consensus on the optimal approach to prevention.

Physical activity is a modifiable and potentially important risk factor for persistent pain and functional decline in older adults involved in an MVC. Promoting physical activity improves outcomes in patients with chronic low back pain and hip fracture, and encouraging a return to normal activities and neck exercises improves outcomes in younger adults with acute neck pain due to MVC (i.e. whiplash) [16–19]. Experimental studies suggest a possible mechanism underlying the benefit of early physical activity following an injury: remaining physically activity during the first two weeks after nerve injury inhibits generation of nociceptive fibers [20]. In contrast, complete inactivity even as brief as one week has profound negative effects on overall health and physical function [21–23], and advising patients to stay in bed following an episode of acute low back pain increases the risk for persistent pain [24]. A recent study of community-dwelling older adults found bed rest to be a stronger predictor of subsequent functional decline than cutting down activities, suggesting that not all types of restricted activity have the same consequences [25]. Understanding the relationship between restricted physical activity during the early aftermath of MVC and long-term outcomes has the potential to inform interventions to prevent the transition from acute to persistent pain.

The objective of this study was to evaluate the relationship between physical activity and persistent pain among older adults involved in an MVC. Specifically, we sought to assess associations between three measures of restricted activity in the first 6 weeks after MVC and persistent pain, pain interference, and decreased functional capacity at 6 months.

## Methods

### Study design and setting

We analyzed data from a multicenter prospective longitudinal study of patients aged 65 years and older who were evaluated in an ED within 24 hours of an MVC. Research assistants (RAs) assessed eligibility and conducted in-person ED interviews. All RAs completed an online course on the protection of human research subjects and study-specific training, including a practice interview prior to enrolling patients. Follow-up surveys were completed via mail or telephone interview 6 weeks and 6 months after the MVC. Patients were consecutively enrolled at eight EDs in four no-fault insurance states (Massachusetts, Michigan, New York, and Florida). No-fault insurance states were chosen to minimize the number of participants for whom on-going legal activity might promote symptom persistence [26]. Institutional Review Board approval was obtained at each study site, and each participant provided written informed consent. Additional details regarding data collection are provided in a related study of outcomes after MVC among younger adults [27].

### Study participants

Patients aged 65 years and older who presented to the ED within 24 hours after an MVC were consecutively screened for enrollment for at least 60 hours a week at each study site. Patients were excluded if they did not speak English; were cognitively impaired as defined by a Six-item Screener score of 3 or less [28, 29]; had a fracture, intracranial injury, thoracic or intra-abdominal injury, spinal cord injury, or another injury that were likely to require hospital admission; or were receiving end of life or comfort care. These inclusion criteria were applied during the ED visit but prior to the final decision to admit or discharge patients; as a result, the sample includes some (*N* = 27) patients who were subsequently admitted to the hospital. None of these patients had life-threatening injuries. Recruitment took place between May 2012 and January 2015.

### Measures

In addition to demographic information, the ED interview assessed pre-MVC disability, pain, sleep, depressive symptoms, and social support, as well as ED pain. Pre-MVC disability was considered present if patients reported at least some difficulty with one or more activities of daily living (ADL). Pre-MVC pain was assessed by asking participants to rate their average overall pain severity (0–10 scale) during the month prior to MVC and was defined as present if pain severity was rated ≥4. Sleep was assessed by asking participants to report their average number of hours of sleep each day during the week prior to MVC, including overnight sleep and naps. Depression was assessed using two questions from the Primary Care Evaluation of Mental Disorders (PRIME-MD), which is a validated tool used to rapidly identify symptoms of depression. Participants were considered depressed if they answered “yes” to either: 1) feeling down, depressed, or hopeless much of the time during the past month, or 2) being bothered by little interest or pleasure in doing things much of the time during the past month [30]. Perceived social support was assessed by summing the scores from three questions from the Medical Outcomes Study Social Support Survey Instrument, and low perceived social support was defined by a social support score in the lowest quartile of scores in this sample [31]. ED pain was assessed by asking patients “Considering all of your pains together, what is the intensity of your pain on a scale of 0 to 10, where 0 means no pain and 10 equals pain as severe as it could possibly be?”; for the 72 (of 164, 44 %) patients who had already received analgesics, patients were asked to recall their pain severity prior to receiving analgesics. Mild pain was defined as a pain score of 1–3; moderate as 4–6; and severe as 7–10.

MVC characteristics assessed during the ED interview included vehicle damage, perception of the MVC as life-threatening, and vehicle speed prior to impact. Participants were asked to characterize the damage to their vehicle as none, minor, moderate, or severe. They were also asked “How life-threatening was your motor vehicle accident? Please rate how close you came to dying on a 0 to 10 scale, where 0 means your life was not threatened at all, and 10 means that you came very close to being killed, or could easily have been killed.” A life-threatening MVC was defined as greater than or equal to the median score of 5. We assessed perceived life-threat from the MVC because perceived threat correlates with the stress exposure from the trauma, which is understood to be an etiologic contributor to persistent pain and may also predispose patients to reduced physical activity [32]. Finally, participants were asked to estimate the speed of their vehicle at the time of collision, and if applicable, the speed of the other vehicle involved. If both vehicles were moving at the time of collision, we used the higher speed in our analyses.

The ED interview also collected baseline measurements of pain interference, ADL difficulty, and physical function. Baseline pain interference, assessed using measures from the Brief Pain Inventory [33], was rated by participants on a 0–10 scale, where 0 is “no interference” and 10 is “complete interference.” Participants were asked how much pain interfered with their general activity, walking ability, sleep, and enjoyment of life during the month before the MVC. Baseline ADL difficulty (0–18 scale) was assessed by asking participants how easily they could complete six different tasks before the MVC: bathing, dressing, transferring from bed to chair, rising from a chair, using the toilet, and eating [34]. Baseline physical function (0–12 scale) was assessed using a validated measure of higher level physical function [35] by asking participants if they could walk a quarter of a mile, walk up a flight of stairs, or lift 10 pounds before the MVC with “no difficulty”, “some difficulty”, or “unable.” For each task, those reporting “no difficulty” for the first question were asked how easily they could walk 1 mile, 2 flights of stairs, or lift 20 pounds before the MVC.

Restricted activity was assessed using three different measures. First, pre-MVC physical activity was assessed in the ED and current physical activity was assessed at 6 weeks using the Physical Activity Scale for the Elderly (PASE), which provides a summary score of activity based on participant responses to 16 questions about activities during the past week [36, 37]. The PASE has been validated in older adults with pain and physical disability [38]. In a general population of individuals aged 65 years and older, PASE scores ranged from 0 to 360 with a median score of 90, a mean score of 100, and standard deviation of 64 [37]. Quartiles of PASE scores were identified to characterize patient activity prior to the MVC. Restricted activity was defined by a ≥25 point decrease in PASE score between baseline and 6 weeks. This cutoff was chosen because it corresponds to 30 minutes of moderate intensity activity and 30 minutes of resistance training daily, which is equivalent to the efficacious intervention described in a recent large clinical trial in older adults [39]. It was also close to both the mean (27) and median (20) decreases in PASE scores within the study sample. Two alternative cutoffs for the PASE score were examined in order to determine if some other threshold of restricted activity demonstrated a significant relationship with 6 month outcomes: 1) any decrease in PASE over the first 6 weeks (i.e. ≥1 point), and 2) a 72 point decrease in PASE, equal to one standard deviation in the change in PASE scores. The other two measures of restricted activity used single-item questions: “During the past two weeks, have you stayed in bed for at least half a day?” and “During the past two weeks, have you cut down on your usual activities as compared to before the accident?” These latter two questions were asked at 6 weeks and generated dichotomous variables based on yes/no responses.

Outcome variables included pain severity, pain interference, increased ADL difficulty, and decreased physical function 6 months after MVC. Pain severity was defined by the patient’s self-reported average MVC-related pain severity (0–10 scale) in the past week, at the time of the 6 month interview. Increased ADL difficulty and functional decline were determined by subtracting the total score at 6 months from the total score at baseline (i.e. pre-MVC physical function and ADL difficulty), with larger values indicating a greater increase in ADL difficulty and decrease in physical function.

### Statistical analyses

We report percentages of patients with restricted activity overall and by subgroups of patients based on sociodemographics, pre-MVC health, and ED pain severity. Chi-squared tests were used to identify statistically significant differences in the frequency of restricted activity. Relationships between each of the three dichotomous measures of restricted activity are characterized using Pearson correlation (a.k.a. the mean square contingency coefficient). We then report 6 month outcomes for patients depending on whether they experienced restricted physical activity 6 weeks after MVC using each of the three measures of restricted activity. The primary outcome was average MVC-related pain in the past week. Secondary outcomes included the percentage of patients with moderate or severe pain (pain score ≥4) at 6 months as well as pain interference, increased ADL difficulty, and functional decline. Potential confounders of the relationship between restricted activity and 6 month outcomes were chosen based on prior research and clinical experience: age, sex, race, education level, pain severity in the ED, pain severity prior to MVC, depressive symptoms, whether or not a lawyer had been hired 6 weeks after MVC, and the estimated speed of the vehicle at the time of impact. Adjusted relationships were determined using the predxcat command, which fits multivariable linear or multivariable logistic regressions and estimates outcomes at the mean value for covariates. All analyses were conducted using Stata 14.1 (StataCorp, College Station, TX).

## Results

**Fig. 1:**
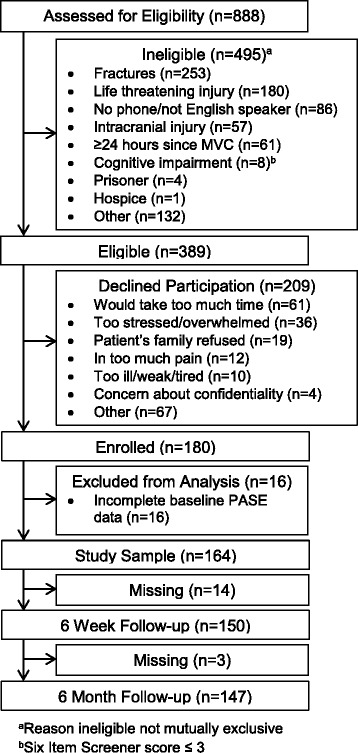
Flow diagram of screening, enrollment, and follow-up

Of 389 eligible patients, 180 (46.2 %) consented to participate and 164 had complete physical activity data in the ED. Sociodemographic characteristics of the individuals who declined participation were similar to those of study participants: most were white (73.0 % vs. 81.1 %), female (58.1 % vs. 61.6 %), and had a similar median age (73 vs. 70). Among the 164 participants with complete physical activity data, 150 (of 164, 91.5 %) completed the 6 week follow-up, and 147 (89.6 %) completed the 6 month follow-up (Fig. 1). Study participants were white (81.1 %), female (61.6 %), aged 65–74 (67.7 %), drivers (78.7 %), and reported moderate (28.9 %) or severe (51.6 %) pain at the time of the ED interview (Table 1). Most reported an overall pre-MVC pain severity of less than 4 (61.0 %), had no ADL difficulty prior to the MVC (80.5 %), and slept an average of 7 or more hours per day (63.5 %). The majority of participants characterized the damage to their vehicle as severe (68.3 %) and considered the MVC life-threatening (53.1 %).

**Table 1:** Characteristics of study participants (*n* = 164)

Characteristic	*n* (%)
Age, years	
65 – 74	111 (67.7)
75 – 84	40 (24.4)
≥85	13 (7.9)
Female	101 (61.6)
Race	
White	133 (81.1)
Black	31 (18.9)
Other	0 (0.0)
College degree^a^	60 (36.8)
Pre-MVC disability^b^	32 (19.5)
Pre-MVC pain ≥4	64 (39.0)
Pre-MVC sleep ≥7 hours	101 (63.5)
Pre-MVC depression	22 (13.4)
Perceived social support^c^	118 (72.0)
Cognitive screener^d^	
4/6	12 (7.5)
5/6	25 (15.5)
6/6	124 (77.0)
Driver	129 (78.7)
ED pain severity	
Mild (1–3)	31 (19.4)
Moderate (4–6)	46 (28.9)
Severe (7–10)	82 (51.6)
Severe damage to car^e^	110 (68.3)
Life-threatening MVC^f^	86 (53.1)
Speed of impact^g^	
0-20 mph	41 (25.0)
30-40 mph	73 (44.5)
50 mph or more	40 (30.5)
Admitted	27 (16.5)
6 month pain severity^h^	
Mild (1–3)	28 (19.1)
Moderate (4–6)	21 (14.3)
Severe (7–10)	26 (17.7)

^a^ *N* = 163

^b^ Defined as at least some difficulty with ≥1 Activity of Daily Living

^c^ Defined as a summary score ≥14 on 3 items from the Medical Outcomes Study Social Support Survey

^d^ Six-item Screener score (0–6), *N* = 161.

^e^ As reported by patient during ED interview, *N* = 161

^f^ Defined as ≥ the median score of 5, as reported by a patient on a scale of 0–10 during ED interview, *N* = 162

^g^ Maximum speed by either patient’s vehicle, or if applicable, the other vehicle involved.

^h^ Average overall pain due to MVC, *N* = 147

Six weeks after MVC, 46.7 % of all study participants had restricted activity based on a ≥25 point decrease in PASE. PASE scores decreased by at least 1 point for the majority of patients (68.0 %), and PASE scores decreased by 1 standard deviation or more (i.e. ≥72 point drop) for 20.7 %. One quarter of patients reported spending at least half a day in bed in the past 2 weeks, and 53.3 % reported cutting down on their usual activity in the past 2 weeks as compared to before the MVC (Table 2). The percentage of patients with a ≥25 point decrease in PASE scores over the first 6 weeks was greater among patients with a low rather than high perceived social support score (59.5 % vs. 41.7 %, *p* = 0.05). Patients with low pre-MVC PASE scores were less likely to have a ≥25 point decrease in PASE score (*p* < 0.001); this relationship was not observed for the other measures of restricted activity. Bed rest in the past two weeks was reported by a larger percentage of patients who reported pre-MVC depressive symptoms vs. those who did not (44.4 % vs. 22.0 %, *p* = 0.05) and by patients with severe pain in the ED vs. those with mild or moderate pain in the ED (36.2 % vs. 14.5 %, *p* < 0.01). Cutting down on activities was reported by a larger percentage of patients who were age 75 or older vs. age 65–74 (67.3 % vs. 46.5 %, *p* = 0.02), patients with low rather than high social support (66.7 % vs. 48.1 %, *p* = 0.04), and patients with moderate or severe pain vs. those with mild pain (63.2 % vs. 25.8 %, *p* < 0.01).

**Table 2:** Percentage of patients with restricted activity by each definition, overall and by participant characteristics (*n* = 150)

	% (95 % CI)		
Characteristic	Decrease in PASE score^a^	Self-reported bed rest	Self-reported cut down on activity
All patients	46.7 (38.9-54.6)	24.7 (18.5-32.1)	53.3 (45.4-61.1)
Age, years			
65 – 74	45.5 (36.1-55.3)	22.8 (15.6-31.9)	46.5 (37.1-56.3)^e^
≥ 75	49.0 (35.4-62.7)	28.6 (17.7-42.6)	67.3 (53.2-78.9)
Sex			
Male	40.4 (28.5-53.5)	24.6 (15.1-37.3)	52.6 (39.8-65.1)
Female	50.5 (40.5-60.5)	24.7 (17.0-34.5)	53.8 (43.6-63.6)
Race			
White	45.3 (36.9-54.0)	25.8 (18.9-34.0)	50.8 (42.2-59.3)
Black	54.5 (34.1-73.5)	18.2 (7.0-39.6)	68.2 (46.6-84.0)
Education			
No College	47.9 (38.0-57.9)	26.6 (18.7-36.4)	51.1 (41.1-61.0)
College	43.6 (31.2-56.9)	20.0 (11.4-32.6)	56.4 (43.1-68.8)
Pre-MVC Activity			
1^st^ quartile	13.9 (5.9-29.3)^e^	30.6 (17.8-47.2)	61.1 (44.6-75.4)
2^nd^ quartile	47.4 (32.3-63.0)	34.2 (21.0-50.4)	50.0 (34.6-65.4)
3^rd^ quartile	53.8 (38.3-68.6)	15.4 (7.1-30.3)	48.7 (33.6-64.0)
4^th^ quartile	70.0 (53.9-82.7)	18.9 (9.3-34.7)	54.1 (38.1-69.2)
Pre-MVC disability^b^			
Yes	36.0 (19.9-56.0)	40.0 (23.0-59.7)	52.0 (33.1-70.4)
No	48.8 (40.2-57.5)	21.6 (15.2-29.7)	53.6 (44.8-62.1)
Pre-MVC pain ≥4			
Yes	49.1 (36.2-62.1)	32.7 (21.7-46.1)	56.4 (43.1-68.8)
No	45.3 (35.6-55.3)	20.0 (13.1-29.2)	51.6 (41.6-61.4)
Sleep			
< 7 hours	47.1 (33.9-60.6)	29.4 (18.6-43.2)	52.9 (39.4-66.1)
≥ 7 hours	45.7 (36.0-55.9)	20.2 (13.3-29.5)	53.2 (43.1-63.0)
Pre-MVC depression			
Yes	50.0 (28.4-71.6)	44.4 (24.0-67.0)^e^	50.0 (28.4-71.6)
No	46.2 (37.9-54.7)	22.0 (15.7-29.8)	53.8 (45.3-62.1)
Social Support^c^			
Yes	41.7 (32.8-51.2)^e^	22.2 (15.4-31.0)	48.1 (38.9-57.5)^e^
No	59.5 (44.3-73.1)	31.0 (18.9-46.3)	66.7 (51.3-79.2)
ED pain severity			
Mild (1–3)	45.2 (28.9-62.6)	9.7 (3.2-26.1)^e^	25.8 (13.5-43.7)^e^
Moderate (4–6)	42.2 (28.8-56.9)	17.8 (9.1-31.7)	64.4 (49.6-76.9)
Severe (7–10)	50.7 (39.1-62.3)	36.2 (25.8-48.1)	62.3 (50.4-72.9)
Damage to car^d^			
None or Mild	66.7 (33.3-88.9)	33.3 (11.1-66.7)	55.6 (25.1-82.3)
Moderate	59.5 (43.2-73.9)	27.0 (15.2-43.3)	56.8 (40.6-71.6)
Severe	40.2 (31.2-50.0)	22.5 (15.5-31.7)	51.0 (41.4-60.5)

^a^ Defined as a ≥25 point decrease in Physical Activity Score for the Elderly (PASE) during the 6 weeks following ED visit

^b^ Defined as at least some difficulty with ≥1 Activity of Daily Living

^c^ Defined as a summary score ≥14 on 3 items from the Medical Outcomes Study Social Support Survey

^d^ As reported by patient during ED interview, N = 161

^e^ p ≤0.05 for comparison of percentage of patients with restricted activity by patient subgroup

Of the 80 patients who reported cutting down on activity at the 6 week interview, only 46 (57.5 %) also had a ≥25 point decrease in PASE score, and 31 (38.8 %) reported bed rest. Of the 70 patients with a ≥25 point decrease in PASE score, 23 (32.9 %) also reported bed rest. Correlations between pairs of these three variables ranged from 0.18 to 0.35.

A PASE score decrease of ≥25 was not associated with pain severity or pain interference at 6 months either before or after adjustment for confounders (Tables 3 and 4). However, patients with a ≥25 point decrease in PASE score had a higher average increase in ADL difficulty than those without a decrease in PASE score (adjusted 0.70 vs. -0.01, *p* = 0.02). (A 1 point decrease in ADL score corresponds to a patient going from have “no difficulty” to “some difficulty,” “some difficulty” to “a lot of difficulty,” or “a lot of difficulty” to “I need help” for one of the six activities assessed.) Neither of the alternative cutoffs for PASE identified subgroups of patients with significantly different adjusted pain severity scores at 6 months: any decrease (*n* = 102) vs. no decrease (*n* = 48) in PASE = 2.04 vs. 2.51, *p* = 0.47; ≥72 point (i.e. one standard deviation) decrease (*n* = 31) vs. <72 point (*n* = 119) decrease = 3.10 vs. 2.00, *p* = 0.14. In adjusted analyses, neither of these alternative cutoffs were associated with either increased ADL difficulty or functional decline at 6 months.

**Table 3:** Unadjusted relationships between three measures of restricted activity at 6 weeks and pain and functional outcomes at 6 months. Values are reported as unadjusted mean (SE)

	Pain and functional outcomes (6 months)				
Measures of restricted activity (6 weeks)	Pain severity^a^	Pain interference with general activity^b^	Pain interference with walking^b^	ADL difficulty^c^	Functional decline^d^
Decrease in PASE score^e^					
Yes	2.85 (0.37)	3.03 (0.36)	2.76 (0.41)	0.72 (0.22)	2.10 (0.45)
No	1.93 (0.35)	2.12 (0.34)	2.57 (0.38)	0.01 (0.20)	0.44 (0.42)
	*p* = 0.07	*p* = 0.07	*p* = 0.74	*p* = 0.02	*p* = 0.01
Self-reported bed rest					
Yes	4.36 (0.49)	4.79 (0.47)	4.79 (0.54)	0.42 (0.31)	2.32 (0.64)
No	1.75 (0.27)	1.86 (0.26)	2.01 (0.30)	0.30 (0.17)	0.88 (0.36)
	*p* < 0.01	*p* < 0.01	*p* < 0.01	*p* = 0.73	*p* = 0.05
Self-reported cut down					
Yes	3.31 (0.33)	3.31 (0.34)	3.38 (0.38)	0.49 (0.21)	2.06 (0.43)
No	1.31 (0.35)	1.70 (0.35)	1.87 (0.39)	0.15 (0.22)	0.33 (0.44)
	*p* < 0.01	*p* < 0.01	*p* = 0.01	*p* = 0.27	*p* = 0.01

^a^ Mean severity of MVC-related pain (0–10 scale) 6 months after MVC, N = 147

^b^ Mean score (0–10 scale) 6 months after MVC, N = 147

^c^ Mean change in score (0–18 scale) from baseline to 6 months after MVC, with higher value indicating greater difficulty with ADLs, N = 146

^d^ Mean change in score (0–12 scale) from baseline to 6 months after MVC, N = 138

^e^ Defined as a ≥ 25 point decrease in Physical Activity Score for the Elderly (PASE) during the 6 weeks following ED visit

**Table 4:** Adjusted relationships between three measures of restricted activity at 6 weeks and pain and functional outcomes at 6 months. Estimates adjusted for age, sex, race, education, pain severity in the ED, pain severity prior to MVC, depressive symptoms, whether a lawyer had been hired 6 weeks after MVC, and the speed of the vehicle at impact (if another vehicle was involved and both vehicles were in motion, the higher speed was used). Values are reported as adjusted mean (SE)

	Pain and functional outcomes (6 months)									
Measures of restricted activity (6 weeks)	Pain severity^a^		Pain interference with general activity^b^		Pain interference with walking^b^		ADL difficulty^c^		Functional decline^d^	
Decrease in PASE score^e^										
Yes	2.54	(0.34)	2.80	(0.33)	2.52	(0.39)	0.70	(0.22)	1.80	(0.46)
No	2.07	(0.32)	2.19	(0.31)	2.64	(0.36)	−0.01	(0.20)	0.57	(0.43)
	*p* = 0.32		*p* = 0.18		*p* = 0.82		*p* = 0.02		*p* = 0.05	
Self-reported bed rest										
Yes	3.56	(0.51)	3.82	(0.49)	3.98	(0.58)	0.12	(0.34)	1.96	(0.71)
No	1.91	(0.27)	2.07	(0.25)	2.17	(0.30)	0.38	(0.18)	0.71	(0.57)
	*p* < 0.01		*p* < 0.01		*p* < 0.01		*p* = 0.52		*p* = 0.21	
Self-reported cut down										
Yes	2.79	(0.35)	2.86	(0.33)	2.98	(0.39)	0.48	(0.22)	1.93	(0.47)
No	1.76	(0.36)	2.07	(0.34)	2.17	(0.40)	0.15	(0.23)	0.37	(0.47)
	*p* = 0.06		*p* = 0.13		*p* = 0.18		*p* = 0.34		*p* = 0.03	

^a^ Mean severity of MVC-related pain (0–10 scale) 6 months after MVC, *N* = 147

^b^ Mean score (0–10 scale) 6 months after MVC, *N* = 147

^c^ Mean change in score (0–18 scale) from baseline to 6 months after MVC, with higher value indicating greater difficulty with ADLs, *N* = 146

^d^ Mean change in score (0–12 scale) from baseline to 6 months after MVC, *N* = 138

^e^ Defined as a ≥ 25 point decrease in Physical Activity Score for the Elderly (PASE) during the 6 weeks following ED visit

In contrast, bed rest was associated with higher pain severity and pain interference at 6 months, and these relationships persisted after adjustment for confounders. Specifically, adjusted average pain severity at 6 months was 3.56 for patients reporting bed rest at the 6 week interview vs. 1.91 for patients who did not report bed rest (*p* < 0.01). After adjustment, among patients reporting bed rest at 6 weeks, 41.6 % reported moderate or severe pain at 6 months; among those not reporting bed rest, only 18.8 % had moderate or severe pain at 6 months (*p* = 0.03). Bed rest was also associated with adjusted average pain interference with general activity (3.82 vs. 2.07, *p* < 0.01) and with walking (3.98 vs. 2.17, *p* < 0.01). Bed rest was not associated with either increased ADL difficulty or functional decline.

Cutting down on activity was associated with greater pain severity and pain interference at 6 months, but the associations between cutting down on activity and pain severity and pain interference did not remain statistically significant after adjustment. After adjustment, among patients reporting cutting down on activities at 6 weeks, 31.0 % reported moderate or severe pain at 6 months, and among those not reporting cutting down on activities, 15.3 % had moderate or severe pain at 6 months (*p* = 0.07). The mean decrease in physical function score over the first 6 months after the MVC was also higher for patients who reported cutting down than for those who did not (adjusted 1.93 vs. 0.37, *p* = 0.03). (A 1 point decrease in physical function score corresponds to a patient going from having “no difficulty” to “some difficulty” or “some difficulty” to “unable” for walking, climbing stairs, or carrying 10 pounds.)

## Discussion

In this sample of older adults evaluated in U.S. EDs after MVC, participants who reported bed rest or cutting down on their physical activity 6 weeks after MVC were more likely to have persistent pain at 6 months than individuals who did not report restricted physical activity. In the same sample, those with a ≥25 point decrease in PASE score between baseline and 6 weeks experienced greater functional decline and difficulty with ADLs 6 months post-MVC. These different assessments of physical activity restriction and change not only differentially predict pain and functional outcomes 6 months post-MVC, but they are also not highly correlated with one another. After adjusting for a priori-selected confounding variables, including sociodemographics, ED pain severity, and depressive symptoms, bed rest remained significantly associated with greater pain severity and pain interference with general activity and walking at 6 months. The 22.8 % absolute difference in the percentage of patients with moderate or severe pain at 6 months among those who reported bed rest vs. those who did not characterizes the magnitude of the difference in pain symptoms across this form of restricted activity.

Somewhat surprisingly, the PASE score, which uses a much more detailed assessment of physical activity, was not associated with persistent pain: patients with a ≥25 point decrease in PASE score at 6 weeks did not have higher mean pain scores at 6 months. However, a decreased PASE score did identify patients who were more likely to experience greater difficulty with ADLs and greater functional decline at 6 months. While the PASE asks individuals to describe how often they completed certain physical activities within the past week (including walking, light exercising, and working/volunteering), the ADL and physical function assessments ask individuals how easily they can take care of themselves and complete basic functional tasks. The observed association between decreased PASE scores and subsequent ADL difficulty and functional decline is consistent with the finding of a large, randomized trial that a physical activity program reduced disability better than a health education program in older adults at-risk for developing disability [39].

Our findings are also consistent with recent work by Gill et al. [25], who report a stronger association between bed rest after illness or injury and long-term outcomes than cutting down on activity and long-term outcomes. Our results also support the hierarchy described by Gill et al., in that 80 patients reported cutting down on activities but only 37 reported bed rest. Our results are novel in that we use decrease in PASE score as one of the definitions of restricted activity and because we examine the relationship between restricted activity and health outcomes following a homogeneous injury mechanism, which reduces variance in outcomes resulting from the type of injury experienced. Additional strengths of this study include that the sample is from multiple sites and has similar demographic characteristics to that of older adults receiving care in U.S. EDs (female 62 % vs. 59 %; white 81 % vs. 80 %) [40].

In our sample, the percentages of patients with a decrease in PASE scores did not vary significantly according to age, education, pre-MVC pain, or symptoms of depression. Specifically, compared to those without symptoms of depression, participants with symptoms of depression prior to the MVC were almost equally likely to have a ≥25 point decrease in PASE scores (50.0 % vs. 46.2 %). In contrast, patients with depressive symptoms were substantially more likely to report spending at least half a day in bed during the past 2 weeks (44.4 % vs. 22.0 %). A possible explanation for this difference is that some of the patients reporting bed rest at 6 weeks were already spending at least half a day in bed prior to the MVC, and that the bed rest question identified a depressed phenotype prior to the MVC. Depressive symptoms were not associated with cutting down on activity, further supporting the findings from the PASE data that depression is not an important determinant of a reduction in activity following the MVC. A decrease in PASE score was less commonly observed among patients with a low level of physical activity prior to the MVC. The most likely explanation for this is that there is a floor effect on PASE scores: if you start out with a low score, it is relatively difficult to further decrease your physical activity score. This floor effect may partly explain why a ≥25 point decrease in PASE score in the first 6 weeks was not associated with pain outcomes at 6 months.

Our findings suggest that assessing physical activity approximately 6 weeks after an MVC may be useful for identifying older adults at increased risk of persistent pain. Whether interventions that promote physical activity after MVC can improve outcomes for older adults is less clear. Exercise improves pain symptoms in patients with chronic musculoskeletal pain [41], and specifically in patients with osteoarthritis of the knee and hip [42]. Older adults’ adherence to exercise regimens for short periods of time, including in the setting of musculoskeletal pain, is generally excellent [43, 44], but the feasibility of a physical activity intervention in older adults experiencing MVC has not been established. Our results indicate that restricted physical activity at 6 weeks is common, with more than half of patients reporting cutting down on activities, but also suggest restricted activity is problematic given the observed associations with pain symptoms and function. Among older adults with a chronic pain condition, periods of increased pain are often followed by reduced physical activity and tend to predispose patients to continued subsequent pain [45]. Although some reduction in physical activity following MVC is probably inevitable for many patients and likely promotes recovery, our data is not sufficiently granular to allow us to distinguish between a protective and a pathologic duration of restricted activity. More work is needed to confirm a causal relationship between restricted activity after MVC and persistent pain and identify the types of interventions that have the greatest impact on outcomes. Given the high prevalence of acute pain in this population and low rates of treatment [14, 46], an intervention to promote physical activity may be more successful if coupled with analgesic therapy.

This study has several limitations. Of the 389 eligible patients, only 46.2 % consented to participate, which raises the concern of whether findings from this sample can be generalized to the population of older adults presenting to the ED after MVC. This concern is mitigated by several observations. First, participants had similar demographic characteristics as non-participants. Second, the percent minority within the study sample (18.9 %) is only slightly lower than the percent in nationally representative samples (24 %) [40]. Third, in reviewing the reasons for non-participation, there are no obvious theoretical reasons why measures of reduced physical activity would be associated with persistent pain and functional decline in patients who agreed to be in the study but not in patients who declined to participate. Forty-four percent of patients received analgesics prior to their ED assessment and so were asked to recall their pain severity prior to receiving analgesics. Although the accuracy of pain recall at one week for patients with low back pain has been found to be excellent [47], the accuracy of patient recall of pain severity in the specific context of this study (i.e. after MVC) has not been studied. Although PASE is specifically designed for and validated in older adults [36, 38], PASE and the other two measures of restricted activity used in this study are based on self-report. Directly measuring activity was beyond the scope of this work but if used in future studies would provide daily measurements of movement and energy expenditure and allow a better understanding of the duration of reduced physical activity after MVC that contributes to persistent pain [48]. Our measurement of depression was based on a dichotomous rather than a scaled response; a continuous variable would have strengthened our characterization of depression. Our primary outcome was MVC-related pain severity at 6 months; the ability of individuals to correctly attribute pain symptoms to a specific cause is difficult to study and not well characterized. Finally, our study is observational and thus cannot definitively establish a causal relationship between restricted physical activity and long-term pain symptoms. In particular, residual and unmeasured confounding may be present.

## Conclusion

Among older adults experiencing MVC, multiple methods of characterizing restricted activity 6 weeks after the MVC were clinically and statistically associated with different adverse health outcomes. Self-reported bed rest and cutting down on activity 6 weeks after the MVC were associated with 6 month pain severity and pain interference; the relationship between bed rest and pain severity and interference remained after adjusting for multiple confounders. PASE scores, which utilize a more detailed assessment of physical activity, were not associated with 6 month pain or pain interference but were associated with greater ADL difficulty and functional decline. These findings provide preliminary support for the hypothesis that promoting physical activity during the early recovery period after MVC may improve recovery among older adults.

### Ethical approval and consent to participate

Institutional Review Board approval was obtained at each study site, and each participant provided written informed consent.

### Availability of data and materials

The dataset supporting the conclusions of this article is available in the Open Science Framework repository [Older Adult CRASH; https://osf.io/efxuk/]. Consistent with the ICMJE position on sharing clinical trial data, we ask that those using the data seek collaboration with our research group, and if collaboration is not possible, that they provide appropriate credit to our group for the collection of this data.

## Abbreviations

ADL: activity of daily living
CI: confidence interval
ED: emergency department
MVC: motor vehicle collision
PASE: physical activity scale for the elderly
PRIME-MD: primary care evaluation of mental disorders
RA: research assistant
SE: standard error
